# Further reductions in the prevalence of obesity in 4-year-old New Zealand children from 2017 to 2019

**DOI:** 10.1038/s41366-022-01095-2

**Published:** 2022-02-25

**Authors:** Lisa Daniels, Barry J. Taylor, Rachael W. Taylor, Barry J. Milne, Justine Camp, Rose Richards, Nichola Shackleton

**Affiliations:** 1 Department of Women’s and Children’s Health, University of Otago, Dunedin, New Zealand; 2 A Better Start National Science Challenge, University of Otago, Dunedin, New Zealand; 3 Department of Medicine, University of Otago, Dunedin, New Zealand; 4 A Better Start National Science Challenge, University of Auckland, Auckland, New Zealand; 5 Centre of Methods and Policy Application in the Social Sciences, University of Auckland, Auckland, New Zealand; 6 Va’a o Tautai, Division of Health Sciences, University of Otago, Dunedin, New Zealand

**Keywords:** Paediatrics, Epidemiology

## Abstract

**Objective:**

To examine whether the prevalence of age- and sex-adjusted BMI at, or above, the 85th, 95th and 99.7th percentiles continues to decline in New Zealand preschool children, over time.

**Methods:**

As part of a national screening programme, 438,972 New Zealand 4-year-old children had their height and weight measured between 2011 and 2019. Age- and sex-adjusted BMI was calculated using WHO Growth Standards and the prevalence of children at, or above, the 85th, 95th, and 99.7th percentiles and at, or below, the 2nd percentile were determined. Log-binomial models were used to estimate linear time trends of ≥85th, ≥95th and ≥99.7th percentiles for the overall sample and separately by sex, deprivation, ethnicity and urban-rural classification.

**Results:**

The percentage of children at, or above, the 85th, 95th and 99.7th percentile reduced by 4.9% [95% CI: 4.1%, 5.7%], 3.5% [95% CI: 2.9%, 4.1%], and 0.9% [95% CI: 0.7%, 1.2%], respectively, between ‘2011/12’ and ‘2018/19’. There was evidence of a decreasing linear trend (risk reduction, per year) for the percentage of children ≥85th (risk ratio (RR): 0.980 [95% CI: 0.978, 0.982]), ≥95th (RR: 0.966 [95% CI: 0.962, 0.969]) and ≥99.7th (RR: 0.957 [95% CI: 0.950, 0.964]) percentiles. Downward trends were also evident across all socioeconomic indicators (sex, ethnicity, deprivation, and urban-rural classification), for each of the BMI thresholds. Larger absolute decreases were evident for children residing in the most deprived compared with the least deprived areas, at each BMI threshold. There appeared to be no consistent trend for the percentage of children ≤2nd percentile.

**Conclusions:**

Reassuringly, continued declines of children with age- and sex-adjusted BMI at, or above, the 85th, 95th and 99.7th percentiles are occurring over time, overall and across all sociodemographic indicators, with little evidence for consistent trends in the prevalence of children at, or below, the 2nd percentile.

## Introduction

Globally, action on high Body Mass Index (BMI) in childhood is recognised as imperative. Paediatric obesity is a major public health concern in New Zealand, with 31% of children and adolescents aged 2–14 years classified as overweight or obese [1]. Obesity disproportionately affects Māori (New Zealand’s indigenous population) and Pasifika children and adolescents, as well as those of lower socioeconomic status [1, 2]. There are also area level differences in child obesity rates in New Zealand, partially driven by differences in obesogenic environments (e.g., access to energy-dense food and leisure facilities) [3–5]. These differences may represent inequities in access to the socioeconomic determinants of health, varying food and physical activity environments, as well as access to care and the quality of care received; all of which influence the risk of increased weight, and the effectiveness of interventions [2, 6].

The development of childhood obesity often starts early in life, with many children considered overweight or obese before they even start school [7]. Once obesity is established, it can be difficult to reverse through intervention [8, 9], as multifaceted changes are required such as changes in diet, activity, environment and sufficient funding to provide intensive high contact interventions with sufficient follow up and support [10]. Early prevention and treatment are key to New Zealand’s policy response [11]. A national preschool screening programme, the B4 School Check (B4SC), is part of that response. Previous data indicate that the prevalence of overweight, obesity and extreme obesity declined between 2010 and 2016 [12]. Importantly, this decline was observed across all sex, ethnicity and deprivation groups, and was not explained by changes in population composition over time. While these initial data seem positive, and could suggest that early prevention and treatment, which are key to New Zealand’s policy response, is working, this analysis only covered a short time period and longer-term monitoring would provide greater confidence that true declines are being observed.

Therefore, the aims of this study were to (i) examine how the prevalence of New Zealand preschool children with BMI z-scores at, or above, the 85th, 95th and 99.7th percentiles has changed from ‘2011/12’ to ‘2018/19’, (ii) examine whether any differences in trends were consistent across sociodemographic characteristics (i.e., sex, ethnicity, deprivation and urban-rural classification), and (iii) determine the prevalence of children with a BMI z-score at, or below, the 2nd percentile.

## Materials/subjects and methods

### Participants

The B4SC is a national programme designed to monitor the health and development of New Zealand’s tamariki (‘children’ in the indigenous language of New Zealand), including growth monitoring [13]. It is available to all families with children turning 4 years of age. The percentage of the eligible population (all 4-year-olds registered with a primary care practitioner) attending the B4SC was estimated by the Ministry of Health as 79% in ‘2011/12’, 80% in ‘2012/13’, 91% in ‘2013/14’, 92% in ‘2014/15’, 92% in ‘2015/16’, 94% in ‘2016/17’, 93% in ‘2017/18’, and 91% in ‘2018/19’ [14]. High coverage of vulnerable groups (i.e., Māori and Pasifika children, children living in areas of high deprivation) is encouraged by linking a portion of District Health Board (DHB) funding for B4SC to help achieve increased coverage for these particular groups. The coverage for Māori children ranged from 71–95% between 2012 and 2019, from 68–92% for Pasifika children, and 80–92% for children living in high deprivation areas [14].

**Fig. 1:**
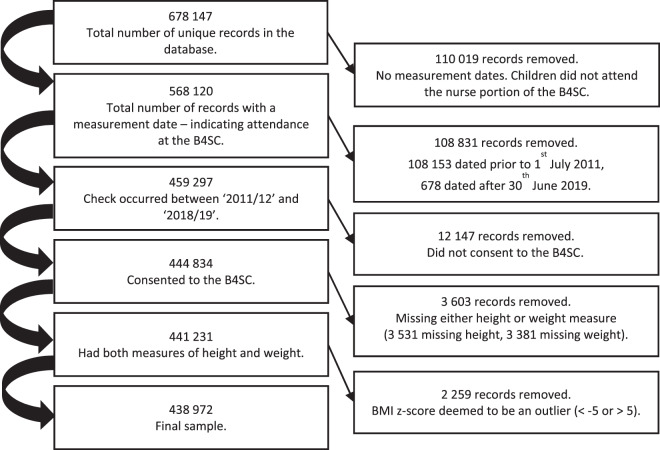
Mapping of exclusions from the B4 School Check database. B4SC B4 School Check, BMI Body Mass Index.

We followed our protocol from our previously published work [12], which includes children who were aged 48–60 months at the time of their B4SC visit. Figure 1 provides an overview of the exclusion criteria. The study was approved by The University of Auckland Human Participants Ethics Committee (Ref: 024418).

### Anthropometric measures

Height and weight were measured by a registered nurse or nurse practitioner. The B4SC handbook instructs that children should be measured wearing light clothing, shoes removed and with equipment stable on a levelled hard surface [15]. Height was measured to the nearest 0.1 cm using a portable stadiometer (either Leicester Height Measure or a SECA 214) and weight to the nearest 0.1 kg using a SECA 862 electronic floor scale or Tanita WB 100 S MA floor scale (or SECA 770 or Tanita HD-351 weighing scale); calibrated at least once every 6 months.

The World Health Organisation (WHO) Anthro Software - STATA ‘igrowup’ package was used to obtain the WHO growth standards including sex-specific BMI-for-age z-scores [16], henceforth referred to as BMI z-score. We used WHO growth standards to ensure continuity with our previously published work [12], because they have been endorsed for New Zealand by the New Zealand Ministry of Health [15], and because this paper concentrates on changes over time within New Zealand children rather than international comparisons.

### Demographic characteristics

Details on demographic characteristics are detailed elsewhere [12]. Briefly, we obtained details on sex, birth month/year and ethnicity by linking to other administrative data in the Statistics New Zealand Integrated Data Infrastructure, a collection of whole-of-population administrative data sources and sample surveys linked at the individual level [17].

#### Ethnicity

Parents could report their child belonging to one or more ethnic groups. Multiple ethnic identification is common in New Zealand [18]. In this sample, 26.6% belonged to two or more of the following major ethnic groups: (i) European; (ii) Māori; (iii) Pacific; (iv) Asian; (v) Middle Eastern, Latin American and African and (vi) Other. Due to their relatively small numbers, Middle Eastern, Latin American and African and Other are not included in stratified analyses. A subset of the analysis considers diversity within Pacific and Asian subgroups, by further subdividing these ethnicity groups into their major subgroups. Pacific was subdivided into Samoan, Tongan, Cook Island Māori and ‘other Pacific’, and Asian into Chinese and Indian subgroups.

#### Socioeconomic deprivation

The relative socioeconomic deprivation of areas was estimated using the New Zealand Index of Deprivation (NZDep) for 2013 [19] for years ‘2011/12’ to ‘2015/16’ and the NZDep for 2018 [20] from years ‘2016/17’ onwards. NZDep uses national census data to measure the level of deprivation for people in small areas containing typically between 100–200 residents. The scale ranges from 1–10 with 1 representing areas of low deprivation and 10 representing areas of high deprivation [20]. Deprivation scores were available for 99.7% of children in the analytic sample.

#### Urban-rural classification

Urban included: main urban, secondary urban and minor urban areas (populations ≥ 1 000) and rural included: rural centre and other rural areas (populations < 999). The urban-rural classification was available for 99.8% of children in the analytic sample.

### Statistical analyses

Data were analysed using Stata version 15 [21]. We created binary variables for children at, or above, the 85th (z-score ≥ 1.036), 95th (z-score ≥ 1.645) and 99.7th (z-score ≥ 2.748) percentiles for age and sex-adjusted BMI as well as for children at, or below, the 2nd (z-score ≤ −2.054) percentile. Deprivation scores (1–10) were collapsed into quintiles (quintile 1: scores 1 and 2, quintile 2: scores 3 and 4, etc.). The mean BMI z-score and the percentage of 4-year-olds within each of the BMI threshold variables (≤2nd percentile, ≥85th percentile, ≥95th percentile, ≥99.7th percentile) for each fiscal year (from ‘2011/12’ to ‘2018/19’) were calculated for the overall sample and separately by sex, deprivation quintile, ethnicity and urban-rural classification. We also calculated the prevalence for subgroups of Pacific and Asian ethnicities.

Log-binomial models were used to estimate linear time trends of ≥85th percentile, ≥95th percentile, and ≥99.7th percentile for the overall sample. These were also estimated separately by sex, deprivation, ethnicity and urban-rural classification, with results expressed as risk ratios per year. An adjusted linear time trend was also calculated for the overall sample for ≥85th percentile, ≥95th percentile, and ≥99.7th percentile adjusting for sex, ethnicity, deprivation and urban or rural residence.

## Results

The sociodemographic characteristics of children by survey year are shown in Table 1. Across all years, a disproportionate percentage (24.1–25.8%) of children resided in areas in the highest quintile of deprivation. Over time, there was an increase in the percentage of children identifying as Asian, from 12.3% in ‘2011/12’ to 20.6% in ‘2018/19’, and a decrease in the percentage that identifies as European, from 73.2% to 64.6%.

**Table 1:** Characteristics of the analytical sample by year.

	2011/12	2012/13	2013/14	2014/15	2015/16	2016/17	2017/18	2018/19
*n*	50,460	50,325	58,020	56,631	57,243	57,057	55,293	53,943
Sex, %								
Female	48.7	48.4	48.6	48.9	48.8	49.0	48.8	48.5
Male	51.3	51.6	51.4	51.1	51.2	51.0	51.2	51.5
Ethnicity^a^, %								
European	73.2	73.5	72.0	70.7	70.1	67.5	66.0	64.6
Māori	27.6	27.6	27.4	27.7	27.1	26.3	25.9	24.9
Pacific	13.8	13.6	14.3	14.6	14.2	14.0	13.7	13.3
Samoan	6.7	6.4	6.8	6.9	6.6	6.6	6.4	6.0
Tongan	3.1	3.1	3.5	3.4	3.5	3.5	3.4	3.2
Cook Island Māori	3.1	3.1	3.2	3.3	3.2	3.2	3.3	3.3
Other Pacific	2.5	2.5	2.6	2.9	2.8	2.7	2.7	2.5
Asian	12.3	12.7	13.7	15.1	16.5	19.1	18.7	20.6
Indian	4.1	4.3	4.5	4.9	5.0	5.8	6.1	6.1
Chinese	3.7	3.8	4.2	4.9	5.8	6.9	6.4	7.3
MELAA	1.8	1.8	1.9	2.0	2.1	2.3	2.2	2.4
Other	2.3	2.3	2.3	2.1	2.1	1.9	1.3	1.1
Area^b^, %								
Urban	87.4	86.9	87.5	87.3	87.5	87.9	86.9	87.0
Rural	12.6	13.1	12.5	12.7	12.5	12.1	13.1	13.0
Deprivation^c^, %								
Q1 (least deprived)	19.5	19.3	19.4	18.9	19.7	19.8	18.2	18.4
Q2	18.5	18.5	18.1	18.7	18.6	18.2	17.8	17.7
Q3	18.2	18.4	18.7	18.2	18.5	18.8	18.6	18.3
Q4	19.0	19.5	19.4	19.5	19.0	19.1	19.6	20.2
Q5 (most deprived)	25.0	24.2	24.5	24.6	24.2	24.1	25.8	25.4
Anthropometry, mean (95% CI)								
Weight (kg)	18.5	18.5	18.5	18.4	18.3	18.3	18.2	18.3
	(18.5, 18.4)	(18.5, 18.5)	(18.5, 18.5)	(18.4, 18.4)	(18.3, 18.4)	(18.3, 18.3)	(18.2, 18.3)	(18.2, 18.3)
Height (cm)	106.3	106.4	106.3	106.0	105.9	105.9	106.0	106.1
	(106.3, 106.4)	(106.3, 106.4)	(106.3, 106.3)	(106.0, 106.1)	(105.9, 105.9)	(105.9, 106.0)	(106.0, 106.1)	(106.1, 106.2)

MELAA: Middle Eastern, Latin American and African.

^a^A child can be classified as belonging to multiple ethnic groups; therefore, the percentages do not equate to 100%.

^b^Urban includes major, secondary and minor urban areas with populations ≥1000, rural areas with populations <999.

^c^Household deprivation categorised using NZDep scale, quintile 1 indicates the lowest level of deprivation and quintile 5 indicates the highest level of deprivation [20].

Table 2 shows the estimated BMI z-scores for each year, by sex, ethnicity, area of residence (urban or rural) and deprivation quintile. There was a larger drop in BMI z-score between ‘2016/17’ (mean BMI z-score = 0.64) and ‘2017/18’ (mean BMI z-score = 0.57) than between any other consecutive years. This reduction in BMI z-score between ‘2016/17’ and ‘2017/18’ is observed across population subgroups investigated. We explored this further and cannot attribute this to error in the data (i.e., outliers or a coding error), or to a change in the weight, height or age distibution of participants (Supplementary A). There is also nothing to suggest national level reforms in measurement practice over these years.

**Table 2:** Mean BMI z-score (95% CI) for the analytical sample by year stratified by sex, ethnicity, area and deprivation^a^.

	2011/12	2012/13	2013/14	2014/15	2015/16	2016/17	2017/18	2018/19
Overall	0.68	0.66	0.66	0.67	0.65	0.64	0.57	0.55
	(0.67, 0.69)	(0.65, 0.67)	(0.66, 0.67)	(0.66, 0.68)	(0.64, 0.66)	(0.63, 0.65)	(0.56, 0.58)	(0.54, 0.56)
Sex								
Female	0.59	0.56	0.58	0.59	0.58	0.57	0.50	0.48
	(0.57, 0.60)	(0.55, 0.58)	(0.56, 0.59)	(0.57, 0.60)	(0.56, 0.59)	(0.56, 0.58)	(0.49, 0.51)	(0.47, 0.49)
Male	0.77	0.75	0.75	0.75	0.73	0.70	0.64	0.61
	(0.76, 0.79)	(0.74, 0.76)	(0.74, 0.76)	(0.73, 0.76)	(0.72, 0.74)	(0.69, 0.72)	(0.63, 0.65)	(0.60, 0.63)
Ethnicity^b^								
European	0.64	0.61	0.61	0.63	0.63	0.63	0.58	0.57
	(0.63, 0.65)	(0.60, 0.62)	(0.61, 0.62)	(0.62, 0.64)	(0.62, 0.64)	(0.62, 0.64)	(0.57, 0.59)	(0.56, 0.58)
Māori	0.88	0.86	0.86	0.87	0.86	0.86	0.78	0.77
	(0.86, 0.90)	(0.84, 0.88)	(0.84, 0.88)	(0.86, 0.89)	(0.84, 0.87)	(0.84, 0.87)	(0.76, 0.80)	(0.75, 0.79)
Pacific	1.18	1.20	1.21	1.17	1.15	1.10	0.99	0.97
	(1.16, 1.21)	(1.17, 1.23)	(1.18, 1.23)	(1.14, 1.19)	(1.12, 1.17)	(1.08, 1.13)	(0.97, 1.02)	(0.94, 1.00)
Samoan	1.24	1.24	1.26	1.23	1.24	1.16	1.08	1.07
	(1.20, 1.28)	(1.20, 1.28)	(1.22, 1.30)	(1.20, 1.27)	(1.20, 1.28)	(1.13, 1.20)	(1.04, 1.11)	(1.03, 1.11)
Tongan	1.33	1.38	1.41	1.32	1.28	1.26	1.10	1.12
	(1.27, 1.39)	(1.32, 1.44)	(1.36, 1.46)	(1.27, 1.37)	(1.24, 1.33)	(1.21, 1.32)	(1.05, 1.15)	(1.07, 1.18)
Cook Island Māori	1.08	1.10	1.08	1.05	1.05	1.01	0.87	0.84
	(1.02, 1.13)	(1.04, 1.15)	(1.03, 1.13)	(1.00, 1.10)	(1.00, 1.10)	(0.96, 1.06)	(0.82, 0.92)	(0.79, 0.89)
Other Pacific	1.09	1.14	1.11	1.06	1.03	1.01	0.92	0.88
	(1.02, 1.15)	(1.07, 1.20)	(1.05, 1.17)	(1.01, 1.11)	(0.98, 1.09)	(0.95, 1.06)	(0.85, 0.98)	(0.82, 0.94)
Asian	0.31	0.24	0.28	0.25	0.26	0.25	0.16	0.14
	(0.28, 0.33)	(0.21, 0.27)	(0.26, 0.31)	(0.23, 0.28)	(0.24, 0.28)	(0.23, 0.27)	(0.13, 0.18)	(0.12, 0.16)
Indian	0.12	0.07	0.10	0.04	0.07	0.03	−0.06	−0.11
	(0.07, 0.18)	(0.02, 0.13)	(0.06, 0.15)	(−0.01, 0.08)	(0.02, 0.11)	(−0.01, 0.07)	(−0.11, −0.02)	(−0.15, −0.07)
Chinese	0.38	0.30	0.32	0.34	0.35	0.33	0.26	0.26
	(0.33, 0.43)	(0.26, 0.35)	(0.28, 0.36)	(0.30, 0.37)	(0.32, 0.39)	(0.30, 0.36)	(0.22, 0.29)	(0.23, 0.29)
Area^c^								
Urban	0.69	0.66	0.67	0.66	0.65	0.63	0.56	0.54
	(0.67, 0.70)	(0.65, 0.67)	(0.66, 0.68)	(0.66, 0.67)	(0.64, 0.66)	(0.62, 0.64)	(0.55, 0.57)	(0.53, 0.55)
Rural	0.66	0.65	0.65	0.69	0.66	0.67	0.62	0.60
	(0.64, 0.69)	(0.62, 0.67)	(0.62, 0.67)	(0.67, 0.71)	(0.64, 0.69)	(0.64, 0.69)	(0.59, 0.64)	(0.58, 0.62)
Deprivation^d^								
Q1 (least deprived)	0.51	0.49	0.48	0.50	0.50	0.50	0.44	0.42
	(0.49, 0.53)	(0.47, 0.51)	(0.46, 0.50)	(0.49, 0.52)	(0.48, 0.52)	(0.48, 0.52)	(0.42, 0.45)	(0.41, 0.44)
Q2	0.56	0.54	0.53	0.55	0.53	0.54	0.46	0.47
	(0.54, 0.58)	(0.52, 0.56)	(0.51, 0.55)	(0.53, 0.57)	(0.52, 0.55)	(0.52, 0.56)	(0.44, 0.48)	(0.45, 0.49)
Q3	0.61	0.60	0.60	0.59	0.60	0.60	0.53	0.50
	(0.59, 0.63)	(0.58, 0.62)	(0.58, 0.62)	(0.57, 0.61)	(0.58, 0.62)	(0.58, 0.62)	(0.51, 0.55)	(0.48, 0.52)
Q4	0.72	0.68	0.71	0.69	0.69	0.66	0.60	0.58
	(0.70, 0.74)	(0.66, 0.70)	(0.69, 0.73)	(0.67, 0.71)	(0.67, 0.71)	(0.64, 0.68)	(0.58, 0.62)	(0.56, 0.60)
Q5 (most deprived)	0.93	0.92	0.92	0.91	0.88	0.84	0.74	0.71
	(0.91, 0.95)	(0.90, 0.94)	(0.90, 0.94)	(0.90, 0.93)	(0.86, 0.90)	(0.82, 0.86)	(0.72, 0.76)	(0.69, 0.73)

^a^BMI z-score was calculated using WHO BMI-for-age growth standards [56].

^b^A child can be classified as belonging to multiple ethnic groups.

^c^Urban includes major, secondary and minor urban areas with populations ≥1 000, rural areas with populations <999.

^d^Household deprivation categorised using NZDep scale, quintile 1 indicates the lowest level of deprivation and quintile 5 indicates the highest level of deprivation [20].

Table 3 shows the percentage of children at, or above, the 85th, 95th and 99.7th percentile over time, as well as estimated unadjusted linear trends. Overall, the percentage of children at, or above, the 85th, 95th and 99.7th percentile reduced by 4.9% [95% CI: 4.1%, 5.7%], 3.5% [95% CI: 2.9%, 4.1%], and 0.9% [95% CI: 0.7%, 1.2%], respectively, between ‘2011/12’ and ‘2018/19’. There was evidence of a decreasing linear trend (risk reduction) for the percentage of children at, or above, the 85th percentile (risk ratio (RR): 0.980 [95% CI: 0.978, 0.982], per year), 95th percentile (RR: 0.966 [95% CI: 0.962, 0.969], per year) and 99.7th percentile (RR: 0.957 [95% CI: 0.950, 0.964], per year). This downward trend remained significant after full adjustment of the models (≥85th percentile RR: 0.974 [95% CI: 0.971, 0.977], ≥95th percentile RR: 0.961 [95% CI: 0.957, 0.965], ≥99.7th percentile RR: 0.954 [95% CI; 0.946, 0.962]). The results for the percentage of children at, or below, the 2nd percentile are presented in Supplementary B.

**Table 3:** Year specific percentage of children at, or above, the 85th percentile, 95th percentile and 99.7th percentile of age and sex-adjusted BMI by sociodemographic characteristics.

≥85th Percentile^a^	2011/12	2012/13	2013/14	2014/15	2015/16	2016/17	2017/18	2018/19	Trend RR^b^
Overall	34.3	33.5	33.3	33.6	32.8	32.1	30.0	29.4	0.980
	(33.9, 34.7)	(33.1, 33.9)	(32.9, 33.6)	(33.3, 34.0)	(32.4, 33.2)	(31.7, 32.5)	(29.6, 30.4)	(29.0, 29.8)	(0.978, 0.982)
Male	38.1	37.2	36.7	36.9	36.2	35.0	33.0	32.2	0.978
	(37.5, 38.7)	(36.6, 37.8)	(36.1, 37.2)	(36.3, 37.4)	(35.7, 36.8)	(34.4, 35.5)	(32.4, 33.5)	(31.7, 32.8)	(0.975, 0.980)
Female	30.3	29.5	29.7	30.3	29.3	29.1	26.9	26.5	0.982
	(29.7, 30.9)	(29.0, 30.1)	(29.1, 30.2)	(29.7, 30.8)	(28.7, 29.8)	(28.6, 29.6)	(26.3, 27.4)	(25.9, 27.0)	(0.980, 0.985)
European	31.9	31.0	30.7	31.5	31.1	31.2	29.2	29.0	0.989
	(31.5, 32.4)	(30.5, 31.4)	(30.2, 31.1)	(31.0, 32.0)	(30.6, 31.5)	(30.8, 31.7)	(28.7, 29.6)	(28.5, 29.4)	(0.987, 0.992)
Māori	41.6	41.2	40.8	41.3	40.8	40.5	38.0	37.7	0.987
	(40.8, 42.5)	(40.4, 42.0)	(40.1, 41.6)	(40.6, 42.1)	(40.0, 41.6)	(39.7, 41.3)	(37.2, 38.8)	(36.9, 38.5)	(0.984, 0.990)
Pacific	53.7	54.0	54.7	53.4	52.0	49.8	45.7	45.8	0.974
	(52.5, 54.9)	(52.9, 55.2)	(53.7, 55.8)	(52.3, 54.5)	(51.0, 53.1)	(48.7, 50.9)	(44.6, 46.8)	(44.7, 47.0)	(0.971, 0.978)
Samoan	56.4	56.0	56.7	55.9	55.4	51.8	48.3	49.3	0.978
	(54.7, 58.0)	(54.3, 57.7)	(55.2, 58.3)	(54.3, 57.4)	(53.9, 57.0)	(50.2, 53.4)	(46.6, 49.9)	(47.6, 51.0)	(0.973, 0.982)
Tongan	57.9	62.0	61.9	58.6	57.4	55.3	49.2	51.9	0.973
	(55.5, 60.4)	(59.6, 64.4)	(59.8, 64.0)	(56.4, 60.8)	(55.2, 59.6)	(53.1, 57.4)	(46.9, 51.4)	(49.5, 54.2)	(0.067, 0.980)
Cook Island Māori	51.2	49.5	50.2	49.1	47.8	46.6	41.3	42.3	0.971
	(48.8, 53.7)	(47.0, 51.9)	(47.9, 52.4)	(46.9, 51.4)	(45.5, 50.1)	(44.3, 48.9)	(39.0, 43.6)	(40.0, 44.6)	(0.964, 0.979)
Other Pacific	49.2	50.4	50.5	48.9	48.4	46.3	43.5	42.2	0.976
	(46.4, 51.9)	(47.6, 53.1)	(47.9, 53.0)	(46.5, 51.4)	(45.9, 50.9)	(43.8, 48.8)	(41.0, 46.0)	(39.6, 44.8)	(0.968, 0.985)
Asian	23.3	21.1	21.8	21.0	20.6	19.6	18.0	17.5	0.963
	(22.3, 24.4)	(20.1, 22.0)	(20.9, 22.7)	(20.2, 21.9)	(19.8, 21.4)	(18.9, 20.3)	(17.2, 18.7)	(16.8, 18.2)	(0.957, 0.969)
Indian	21.0	19.5	20.6	18.2	19.5	17.0	16.1	15.6	0.958
	(19.3, 22.8)	(17.8, 21.1)	(19.0, 22.1)	(16.7, 19.6)	(18.0, 20.9)	(15.7, 18.3)	(14.9, 17.4)	(14.3, 16.8)	(0.946, 0.970)
Chinese	23.0	19.8	20.4	21.8	20.8	19.3	17.8	17.2	0.966
	(21.1, 24.9)	(18.0, 21.5)	(18.8, 22.0)	(20.3, 23.3)	(19.5, 22.2)	(18.0, 20.5)	(16.6, 19.1)	(16.0, 18.4)	(0.955, 0.977)
Urban^c^	34.6	33.6	33.5	33.8	32.8	32.1	29.8	29.3	0.978
	(34.1, 35.0)	(33.2, 34.1)	(33.1, 33.9)	(33.3, 34.2)	(32.4, 33.2)	(31.6, 32.5)	(29.4, 30.3)	(28.9, 29.7)	(0.976, 0.980)
Rural^d^	32.7	32.3	31.4	32.9	33.1	32.5	31.0	30.4	0.993
	(31.5, 33.8)	(31.2, 33.5)	(30.3, 32.4)	(31.8, 34.0)	(32.0, 34.2)	(31.4, 33.6)	(29.9, 32.1)	(29.3, 31.5)	(0.988, 0.998)
Deprivation^e^ Q1	27.1	26.1	25.5	26.2	26.0	25.7	24.4	23.1	0.983
	(26.2, 28.0)	(25.3, 27.0)	(24.7, 26.3)	(25.3, 27.0)	(25.2, 26.8)	(24.9, 26.5)	(23.5, 25.2)	(22.3, 24.0)	(0.978, 0.989)
Deprivation^e^ Q2	29.5	28.4	27.8	28.8	27.9	28.3	25.3	26.1	0.984
	(28.6, 30.5)	(27.5, 29.3)	(26.9, 28.7)	(27.9, 29.6)	(27.1, 28.8)	(27.5, 29.2)	(24.4, 26.2)	(25.2, 27.0)	(0.979, 0.989)
Deprivation^e^ Q3	31.9	31.0	30.7	30.9	30.4	30.6	28.1	26.8	0.980
	(30.9, 32.9)	(30.0–31.9)	(29.8, 31.5)	(30.0, 31.8)	(29.6, 31.3)	(29.8, 31.5)	(27.3, 29.0)	(25.9, 27.7)	(0.976, 0.985)
Deprivation^e^ Q4	36.1	35.0	35.1	34.9	34.7	33.3	31.7	30.9	0.980
	(35.1, 37.1)	(34.0–35.9)	(34.3, 36.0)	(34.0, 35.8)	(33.8, 35.6)	(32.4, 34.2)	(30.9, 32.6)	(30.1, 31.8)	(0.976, 0.984)
Deprivation^e^ Q5	43.9	43.9	43.8	44.1	42.5	40.5	37.2	37.1	0.974
	(43.1, 44.8)	(43.0–44.8)	(43.0, 44.7)	(43.3, 44.9)	(41.7, 43.4)	(39.7, 41.3)	(36.4, 38.0)	(36.3, 37.9)	(0.971, 0.977)

≥95th Percentile^f^	2011/12	2012/13	2013/14	2014/15	2015/16	2016/17	2017/18	2018/19	Trend RR^b^
Overall	16.1	15.6	15.3	15.5	14.8	14.0	12.9	12.5	0.966
	(15.7, 16.4)	(15.3, 15.9)	(15.0, 15.6)	(15.2, 15.8)	(14.5, 15.1)	(13.7, 14.2)	(12.6, 13.1)	(12.3, 12.8)	(0.962, 0.969)
Male	18.5	17.9	17.4	17.6	17.0	15.7	14.4	14.1	0.962
	(18.0, 18.9)	(17.5, 18.4)	(17.0, 17.8)	(17.2, 18.1)	(16.6, 17.4)	(15.3, 16.1)	(14.0, 14.8)	(13.7, 14.5)	(0.958, 0.966)
Female	13.5	13.1	13.0	13.2	12.5	12.2	11.2	10.9	0.970
	(13.1, 14.0)	(12.6, 13.5)	(12.6, 13.4)	(12.8, 13.6)	(12.1, 12.9)	(11.8, 12.6)	(10.8, 11.6)	(10.5, 11.2)	(0.966, 0.975)
European	13.7	12.9	12.7	13.4	12.9	12.4	11.3	11.3	0.976
	(13.3, 14.0)	(12.6, 13.3)	(12.3, 13.0)	(13.0, 13.7)	(12.6, 13.3)	(12.1, 12.7)	(11.0, 11.7)	(11.0, 11.6)	(0.972, 0.980)
Māori	20.7	20.5	20.1	20.5	19.8	19.2	17.2	17.6	0.975
	(20.0, 21.3)	(19.9, 21.2)	(19.4, 20.7)	(19.9, 21.1)	(19.1, 20.4)	(18.5, 19.8)	(16.5, 17.8)	(17.0, 18.3)	(0.970, 0.980)
Pacific	31.7	32.5	31.7	30.0	29.8	27.4	24.9	25.0	0.961
	(30.6, 32.7)	(31.3, 33.6)	(30.7, 32.7)	(29.0, 31.0)	(28.8, 30.7)	(26.4, 28.3)	(24.0, 25.9)	(24.0, 26.0)	(0.956, 0.966)
Samoan	32.7	33.2	32.9	31.5	32.5	29.2	26.8	27.1	0.969
	(31.2, 34.3)	(31.6, 34.9)	(31.5, 34.4)	(30.1, 33.0)	(31.0, 34.0)	(27.7, 30.6)	(25.4, 28.3)	(25.6, 28.6)	(0.962, 0.977)
Tongan	37.2	39.9	38.9	34.9	35.2	31.2	28.3	30.4	0.956
	(34.8, 39.6)	(37.5, 42.3)	(36.7, 41.0)	(32.8, 37.0)	(33.1, 37.3)	(29.2, 33.3)	(26.3, 30.3)	(28.2, 32.6)	(0.947, 0.966)
Cook Island Māori	28.4	27.2	27.2	27.0	27.0	25.7	20.3	21.2	0.959
	(26.2, 30.6)	(25.0, 29.5)	(25.1, 29.2)	(25.0, 29.0)	(24.9, 29.0)	(23.7, 27.7)	(18.5, 22.1)	(19.3, 23.1)	(0.947, 0.971)
Other Pacific	28.7	30.7	28.5	26.7	26.2	24.5	23.2	22.7	0.959
	(26.2, 31.2)	(28.2, 33.3)	(26.2, 30.8)	(24.5, 28.8)	(24.1, 28.4)	(22.3, 26.7)	(21.1, 25.4)	(20.5, 25.0)	(0.946, 0.973)
Asian	10.9	10.1	9.5	8.9	8.2	8.2	7.4	6.9	0.940
	(10.1, 11.7)	(9.4, 10.8)	(8.9, 10.1)	(8.3, 9.5)	(7.6, 8.7)	(7.7, 8.7)	(6.9, 7.9)	(6.5, 7.4)	(0.930, 0.950)
Indian	10.7	10.6	9.9	8.7	9.1	8.2	7.7	7.1	0.943
	(9.3, 12.0)	(9.3, 11.9)	(8.8, 11.1)	(7.6, 9.7)	(8.0, 10.1)	(7.3, 9.1)	(6.8, 8.6)	(6.3, 8.0)	(0.925, 0.960)
Chinese	10.0	8.3	7.8	8.3	6.7	6.6	6.1	5.7	0.929
	(8.7, 11.4)	(7.0, 9.5)	(6.7, 8.9)	(7.3, 9.3)	(5.8, 7.5)	(5.9, 7.4)	(5.3, 6.9)	(5.0, 6.4)	(0.910, 0.948)
Urban^c^	16.4	15.9	15.6	15.7	15.0	14.1	13.0	12.6	0.963
	(16.1, 16.8)	(15.5, 16.2)	(15.3, 15.9)	(15.4, 16.0)	(14.7, 15.3)	(13.8, 14.4)	(12.7, 13.3)	(12.3, 12.9)	(0.960, 0.966)
Rural^d^	13.8	13.5	13.1	14.1	13.6	13.2	12.2	12.1	0.984
	(12.9, 14.6)	(12.6, 14.3)	(12.3, 13.8)	(13.3, 14.9)	(12.8, 14.4)	(12.4, 14.0)	(11.5, 13.0)	(11.4, 12.9)	(0.975, 0.993)
Deprivation^e^ Q1	10.6	9.8	9.6	9.7	9.6	8.7	8.3	8.3	0.967
	(10.0, 11.2)	(9.2, 10.4)	(9.1, 10.2)	(9.1, 10.3)	(9.0, 10.1)	(8.2, 9.2)	(7.8, 8.8)	(7.7, 8.8)	(0.958, 0.976)
Deprivation^e^ Q2	12.6	11.8	11.1	11.7	10.9	11.1	9.9	9.4	0.966
	(11.9, 13.2)	(11.1, 12.4)	(10.5, 11.7)	(11.1, 12.3)	(10.3, 11.5)	(10.5, 11.7)	(9.3, 10.5)	(8.9, 10.0)	(0.958, 0.975)
Deprivation^e^ Q3	13.4	13.7	13.1	13.5	13.0	12.9	11.7	10.6	0.972
	(12.7, 14.1)	(13.0, 14.4)	(12.4, 13.7)	(12.9, 14.2)	(12.3, 13.6)	(12.3, 13.6)	(11.1, 12.4)	(10.0, 11.2)	(0.964, 0.980)
Deprivation^e^ Q4	17.1	16.4	16.8	16.7	16.2	15.4	13.9	13.6	0.968
	(16.4, 17.9)	(15.7, 17.1)	(16.1, 17.5)	(16.0, 17.4)	(15.5, 16.9)	(14.7, 16.1)	(13.3, 14.6)	(12.9, 14.2)	(0.962, 0.975)
Deprivation^e^ Q5	24.1	23.8	23.3	23.3	22.4	20.2	18.1	18.4	0.957
	(23.4, 24.9)	(23.1, 24.6)	(22.6, 24.0)	(22.6, 24.0)	(21.7, 23.1)	(19.5, 20.8)	(17.5, 18.7)	(17.7, 19.0)	(0.952, 0.962)

≥99.7th Percentile	2011/12	2012/13	2013/14	2014/15	2015/16	2016/17	2017/18	2018/19	Trend RR^b^
Overall	3.4	3.3	3.2	3.0	2.9	2.9	2.6	2.5	0.957
	(3.3, 3.6)	(3.1, 3.4)	(3.1, 3.4)	(2.9, 3.2)	(2.8, 3.1)	(2.7, 3.0)	(2.4, 2.7)	(2.4, 2.6)	(0.950, 0.964)
Male	4.0	3.7	3.7	3.4	3.2	3.2	3.0	2.8	0.953
	(3.7, 4.2)	(3.5, 4.0)	(3.5, 3.9)	(3.2, 3.6)	(3.0, 3.4)	(3.0, 3.4)	(2.8, 3.2)	(2.6, 3.0)	(0.943, 0.962)
Female	2.8	2.7	2.7	2.6	2.6	2.6	2.2	2.2	0.964
	(2.6, 3.1)	(2.5, 2.9)	(2.5, 2.9)	(2.4, 2.8)	(2.4, 2.8)	(2.4, 2.8)	(2.0, 2.3)	(2.0, 2.3)	(0.953, 0.975)
European	2.3	2.2	2.2	2.1	2.1	2.0	1.8	1.8	0.968
	(2.2, 2.5)	(2.0, 2.3)	(2.0, 2.3)	(1.9, 2.2)	(2.0, 2.3)	(1.8, 2.1)	(1.7, 1.9)	(1.7, 2.0)	(0.958, 0.979)
Māori	4.7	4.5	4.2	4.4	4.1	4.4	3.6	3.8	0.970
	(4.4, 5.1)	(4.2, 4.9)	(3.9, 4.5)	(4.1, 4.7)	(3.8, 4.5)	(4.0, 4.7)	(3.3, 4.0)	(3.5, 4.1)	(0.959, 0.982)
Pacific	9.6	9.4	8.7	8.1	7.8	7.8	7.0	6.7	0.949
	(8.9, 10.3)	(8.7, 10.1)	(8.1, 9.3)	(7.5, 8.6)	(7.3, 8.4)	(7.2, 8.4)	(6.4, 7.6)	(6.1, 7.2)	(0.937, 0.960)
Samoan	9.8	10.4	9.1	8.8	9.2	8.4	8.1	7.6	0.963
	(8.8, 10.8)	(9.3, 11.4)	(8.2, 10.0)	(7.9, 9.7)	(8.2, 10.1)	(7.5, 9.3)	(7.2, 9.0)	(6.7, 8.5)	(0.947, 0.979)
Tongan	12.0	11.5	12.2	10.0	9.2	10.3	8.1	8.6	0.946
	(10.4, 13.6)	(9.9, 13.1)	(10.7, 13.6)	(8.7, 11.4)	(7.9, 10.5)	(9.0, 11.6)	(6.9, 9.3)	(7.3, 10.0)	(0.925, 0.966)
Cook Island Māori	6.9	7.9	6.3	6.6	6.9	6.4	4.8	5.7	0.957
	(5.7, 8.2)	(6.6, 9.3)	(5.2, 7.4)	(5.5, 7.7)	(5.7, 8.0)	(5.2, 7.5)	(3.8, 5.8)	(4.6, 6.7)	(0.930, 0.984)
Other Pacific	10.2	8.9	7.0	6.8	6.2	6.9	6.7	6.0	0.937
	(8.5, 11.9)	(7.3, 10.4)	(5.7, 8.3)	(5.6, 8.1)	(5.0, 7.4)	(5.7, 8.2)	(5.4, 8.0)	(4.8, 7.3)	(0.910, 0.965)
Asian	2.5	2.3	2.5	2.0	1.7	1.7	1.7	1.7	0.936
	(2.1, 2.9)	(2.0, 2.7)	(2.1, 2.8)	(1.7, 2.3)	(1.5, 2.0)	(1.5, 2.0)	(1.5, 2.0)	(1.4, 1.9)	(0.915, 0.958)
Indian	3.3	3.2	3.3	2.4	2.4	2.0	2.6	2.1	0.933
	(2.5, 4.1)	(2.4, 3.9)	(2.6, 4.0)	(1.8, 3.0)	(1.9, 3.0)	(1.6, 2.5)	(2.0, 3.1)	(1.6, 2.5)	(0.901, 0.967)
Chinese	1.6	1.1	1.4	1.3	1.2	1.1	0.9	1.0	0.942
	(1.0, 2.2)	(0.6, 1.6)	(0.9, 1.9)	(0.9, 1.7)	(0.8, 1.5)	(0.8, 1.4)	(0.6, 1.3)	(0.7, 1.3)	(0.893, 0.993)
Urban^c^	3.6	3.4	3.4	3.1	3.0	3.0	2.7	2.6	0.957
	(3.4, 3.7)	(3.2, 3.6)	(3.2, 3.5)	(2.9, 3.2)	(2.9, 3.2)	(2.8, 3.1)	(2.6, 2.9)	(2.5, 2.8)	(0.949, 0.964)
Rural^d^	2.5	2.2	2.1	2.6	2.2	2.1	1.7	1.9	0.963
	(2.1, 2.9)	(1.8, 2.6)	(1.8, 2.5)	(2.3, 3.0)	(1.9, 2.6)	(1.8, 2.4)	(1.4, 2.0)	(1.6, 2.2)	(0.939, 0.987)
Deprivation^e^ Q1	1.5	1.4	1.1	1.3	1.2	1.2	1.0	1.0	0.950
	(1.2, 1.7)	(1.2, 1.7)	(0.9, 1.3)	(1.0, 1.5)	(1.0, 1.4)	(1.0, 1.4)	(0.8, 1.2)	(0.8, 1.2)	(0.925, 0.977)
Deprivation^e^ Q2	2.0	1.8	2.0	1.7	1.6	1.6	1.5	1.7	0.966
	(1.7, 2.3)	(1.5, 2.0)	(1.7, 2.2)	(1.5, 2.0)	(1.3, 1.8)	(1.4, 1.9)	(1.2, 1.7)	(1.4, 1.9)	(0.944, 0.989)
Deprivation^e^ Q3	2.4	2.5	2.5	2.4	2.5	2.3	2.2	2.0	0.977
	(2.1, 2.7)	(2.2, 2.9)	(2.2, 2.7)	(2.1, 2.7)	(2.2, 2.8)	(2.0, 2.6)	(2.0, 2.5)	(1.8, 2.3)	(0.958, 0.997)
Deprivation^e^ Q4	3.9	3.6	3.7	3.3	3.3	3.3	2.7	2.8	0.953
	(3.5, 4.3)	(3.2, 4.0)	(3.3, 4.0)	(3.0, 3.7)	(3.0, 3.7)	(3.0, 3.6)	(2.4, 3.0)	(2.5, 3.1)	(0.937, 0.968)
Deprivation^e^ Q5	6.4	6.1	6.0	5.6	5.4	5.3	4.6	4.3	0.948
	(6.0, 6.8)	(5.7, 6.5)	(5.6, 6.4)	(5.2, 6.0)	(5.0, 5.8)	(4.9, 5.7)	(4.3, 4.9)	(4.0, 4.7)	(0.938, 0.958)

Percentages expressed as mean (95% CI).

^a^≥85th percentile also includes those in the ≥95th percentile and ≥99.7th percentile.

^b^Trend expressed as a risk ratio (RR). RR represent the average change in prevalence per year (year as the continuous variable). These are relative measures of change.

^c^Includes major, secondary and minor urban areas with populations ≥1000.

^d^Includes areas with populations <999.

^e^Household deprivation categorised using NZDep scale, quintile 1 indicates the lowest level of deprivation and quintile 5 indicates the highest level of deprivation [20].

^f^≥95th percentile also includes those in the ≥99.7th percentile.

Across all major ethnic groups, there was a downward trend in the average percentage of children at, or above, the 85th, 95th and 99.7th percentiles over time (Table 3). Reductions in the percentage of children ≥85th percentile were largest for Pacific (7.9% [95% CI: 5.5%, 10.0%]) and Asian (5.8% [95% CI: 4.1%, 7.6%]) ethnicities, and were smaller for European (2.9% [95% CI: 2.1%, 3.9%]) ethnicity, compared to the overall population (4.9% [95% CI: 4.1%, 5.7%]). Reductions in the percentage of children at, or above, the 95th and 99.7th percentiles were larger for Pacific children (≥95th percentile: 6.7% [95% CI: 4.6%, 8.7%], ≥99.7th percentile: 2.9% [95% CI: 1.7%, 4.2%]) compared to the overall population (≥95th percentile: 3.6% [95% CI: 2.9%, 4.1%], ≥99.7th percentile: 0.9% [95% CI: 0.7%, 0.9%]). Relative to the initial prevalence (previous year), those classified as of Asian ethnicity had the largest decrease in the percentage of children ≥85th percentile (RR: 0.963 [95% CI: 0.957, 0.969], per year), ≥95th percentile (RR: 0.940 [95% CI: 0.930, 0.950], per year), and ≥99.7th percentile (RR: 0.936 [95% CI: 0.915, 0.958], per year). Downward trends were also evident across all ethnic subgroups for each of the ≥85th, ≥95th and ≥99.7th percentile BMI thresholds.

There was a greater relative decrease in the number of children at, or above, the 85th percentile for those residing in urban areas compared to children residing in rural areas (urban RR: 0.978 [95% CI: 0.976, 0.980], rural RR: 0.993 [95% CI: 0.988, 0.998]), and ≥95th percentile (urban RR: 0.963 [95% CI: 0.960, 0.966], rural RR: 0.984 [95% CI: 0.975, 0.993]). Absolute decreases in the percentage of children at, or above, the 85th percentile (urban: 5.3% [95% CI: 4.4%, 6.1%], rural: 2.3% [95% CI: 0.0%, 4.5%]) and ≥95th percentile (urban: 3.8% [95% CI: 3.2%, 4.5%], rural: 1.7% [95% CI: 0.0%, 3.5%]) also tended to be larger for children residing in urban areas. For the ≥99.7th percentile, relative (urban RR: 0.957 [95% CI: 0.949, 0.964], rural RR: 0.963 [95% CI: 0.939, 0.987]) and absolute decreases (urban: 1.0 % [95% CI: 0.6%, 1.2%], rural: 0.6% [95% CI: −0.1%, 1.3%]) were similar over time for rural and urban children. The larger decreases for children residing in urban areas over time means that by ‘2018/19’, there was very little difference between those residing in urban and rural areas in the percentage of children in the BMI thresholds of ≥85th and ≥95th percentiles. However, the percentage of children in the ≥99.7th percentile was consistently (across all years) more likely to be those residing in urban areas.

**Fig. 2:**
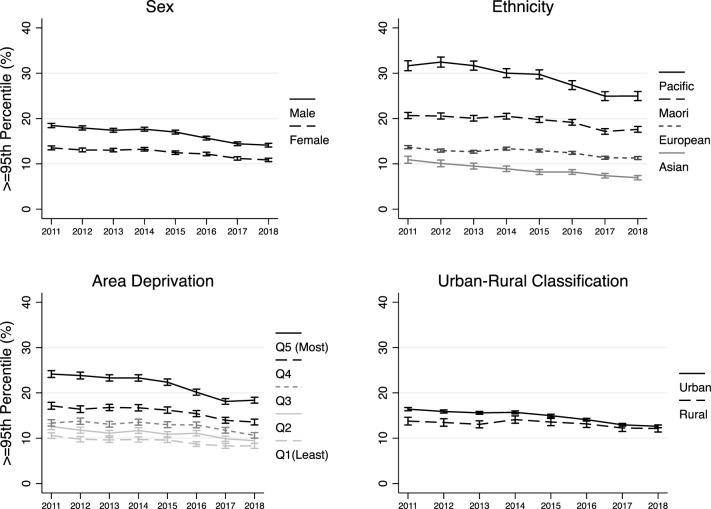
Trends in the percentage of children at, or above, the 95th percentile. Panels show yearly trends by sex, ethnicity, area deprivation and urban-rural classification.

Relative changes in BMI percentiles were similar across deprivation quintiles. Over the 8 year period there was a slightly greater relative decrease in the percentage of children at, or above, the 85th percentile for those residing in the most deprived areas (deprivation Q5) (RR: 0.974 [95% CI: 0.971, 0.977]) compared with those residing in the least deprived areas (deprivation Q1) (RR: 0.983 [95% CI: 0.978, 0.989]). There were larger absolute decreases in each of the BMI percentiles for children residing in the most deprived (Q5) areas compared with those living in the least deprived (Q1) areas (≥85th percentile, Q5: 6.8% [95% CI: 5.2%, 8.5%]; Q1: 4.0% [95% CI: 2.2%, 5.7%], ≥95th percentile, Q5: 5.7% [95% CI: 4.4%, 7.2%]; Q1 2.3% [95% CI: 1.2%, 3.5%], and ≥99.7th percentile, Q5: 2.1% [95% CI: 1.3%, 2.8%]; Q1: 0.5% [95% CI: 0.0%, 0.9%]. The trends over time for the percentage of children at, or above, the 95th percentile by sex, ethnicity, deprivation and urban-rural classification are presented in Fig. 2.

## Discussion

This paper reports decreasing rates of overweight, obesity and extreme obesity in New Zealand 4-year-old children from 2012 to 2019, extending our previous findings demonstrating decreasing rates up to 2016 [12]. Importantly, we found continued declines in the prevalence both across the board and by all indicators of sociodemographic characteristics examined - sex, area level deprivation, ethnicity and urban-rural classification. Importantly, we observed a narrowing in socioeconomic disparities, showing more pronounced decreases in prevalence above each of the BMI thresholds for children residing in the most deprived areas compared with those living in the least deprived areas. Reassuringly, there was little evidence for consistent trends over time for the prevalence of underweight (those at, or below, the 2nd percentile for age- and sex- adjusted BMI), though we note a considerable increase in the last 2 years of data in those from the lowest two deprivation quintiles.

Our finding of a continued decrease in prevalence are in line with several other studies in high income countries where stabilising or decreasing trends in the prevalence of young children classified as overweight and/or obese have been reported [22–35]. Reducing inequities in the prevalence of high BMI values is a major health goal of many countries, but unfortunately, differences in trends according to ethnicity and/or socioeconomic deprivation (SES) are not always reported. In contrast to our study, several studies in young children from the USA have reported that while the prevalence of obesity is generally declining in most ethnic groups, it is not consistent across all, with some ethnic groups showing continued increases in obesity prevalence [29, 36].

Our study shows that while there are more children at, or above, the 95th percentile residing in areas of high compared with low deprivation overall, all levels of deprivation are showing consistent declines in the prevalence of children above this BMI percentile. Similar findings have been reported in other high income countries [29, 31, 37], but not all [34]. Data from the UK National Child Measurement Programme show that inequalities continue to widen among young 4-year-old children with obesity prevalence increasing over time in children living in the most deprived compared to the least deprived areas [34]. While there is a known inverse association between obesity and SES in the developed world, our findings demonstrate that the gap (between the most and least deprived) is lessening in our young New Zealand children, with a higher reduction in the prevalence of children above this age- and sex-adjusted BMI thresholds living in the most deprived areas.

Very few studies report urban-rural differences in the prevalence of overweight and obesity over time [38], and those that do, report conflicting findings. We reported larger decreases in age- and sex-adjusted BMI over time in urban children, which resulted in a very similar prevalence of children at, or above, the 85th and 95th percentiles for both urban and rural children by ‘2018/19’. In contrast, there were larger decreases in the prevalence of obesity for rural Spanish children aged 2–5 years than urban Spanish children, between 2006 and 2016, although urban Spanish children overall had a higher prevalence of obesity [31]. Recent Australian data support our New Zealand findings in part, also showing declines in the prevalence of high BMI z-score (>+1 SD) in children (aged 1–3.5 years) living in major cities (urban) [35]. However, unlike our New Zealand data, these authors report that the prevalence of high BMI was increasing in those living rurally [35].

What is producing these marked declines in the prevalence of high BMI, particularly across all groups examined, is uncertain. Others have suggested that contributions towards declines in overweight and obesity in preschool children could include: efforts to focus on public health interventions and initiatives (promoting healthy eating and physical activity) [25, 29, 30], increased parental education [29, 33], decreasing unemployment rates [33], decreased maternal smoking during pregnancy [23], increased breastfeeding prevalence [23] and increasing proportion of mothers born overseas where lower population BMIs are present [35]. A recent analysis of New Zealand data suggests that continued reductions in maternal smoking during pregnancy may be at least playing a part in the decline found in our study [39]. Another factor possibly contributing towards this shift is New Zealand’s approach to affordable access to early childhood education (ECE) for all New Zealand families [40], where all 3 and 4-year-old children are entitled to 20 h of free ECE participation, since ‘2007/08’ [41]. As part of Te Whāriki (New Zealand’s early childhood curriculum) promoting opportunities for physical activity and nutrition are forefront in the protection of children’s wellbeing [42]. A study investigating New Zealand licensed ECE centres suggested that children spend most of their time in active play and have very little or no screen time while in early childhood care [43]. Alternatively, the declines observed in this age group may reflect societal changes (attitudes and awareness of obesity prevention across the population) [40]. The overall reducing trends in overweight/obesity and across sociodemographic subgroups in this study appears to suggest that this subpopulation (4-year-old New Zealand children) is in the ‘fourth stage’ of the ‘obesity transition’, where the obesity epidemic starts to turn towards declining prevalence, as outlined by Jaacks et al. [44].

We observed no consistent trends in the prevalence of children with low BMI values (≤2nd percentile), supporting earlier work in several countries [37, 45]. An exception to this evidence is a recent study by Zeglen et al. [33] that reported an overall decrease in the prevalence of underweight in Polish children (3–7 years). Of note, is the concerning doubling in the prevalence of 4-year-old children at, or below, the 2nd percentile living in areas of high deprivation compared with lower deprivation quintiles, between ‘2016/17’ and ‘2018/19’ that requires further investigation.

Our study results have been intentionally reported in percentiles and not labelled as ‘risk-of-overweight’, ‘obese’ or ‘extremely obese’ due to ongoing debate about the appropriateness of different BMI thresholds for predicting body fat across different ethnic groups in New Zealand [46–48]. However, at the population level, BMI still remains a useful predictor of body fat and health outcomes with risk continuously increasing with positive BMI z-scores [49–52]. While the use of percentiles instead of cut-points may create some misclassification [53], our objective was to compare with our previously published data using percentiles [12].

The present study has major strengths in that all measures of weight and height were direct measures and not self-reported. Registered nurses conducting B4SC’s followed a standard protocol for anthropometric measurements [15]. We report eight consecutive years of data which is generalisable at the national level in New Zealand. As the percentage of the eligible population completing the B4SC was high over the analytical years (79–94%), the results are an almost complete representative sample of New Zealand 4-year-old children. However, our study also has some limitations. Not every eligible child in New Zealand completes a B4SC, and previous research has reported that children of lower SES or poor health are less likely to get a B4SC [54]. However, coverage rates of our more vulnerable population groups have improved over the years reported. Our study reports trends in the prevalence of age- and sex-adjusted BMI for 4-year-old children in New Zealand, there is currently no similar large data reporting trends in younger (<4 years) or older (>4 years) children. Finally, as this was a descriptive study, we did not assess the reasons for the declining prevalence in this population group.

In conclusion, this nationally representative study of 4-year-old New Zealand children shows a continued decline in children with an age- and sex-adjusted BMI at, or above, the 85th, 95th and 99.7th percentiles, overall and across all sociodemographic indicators, with little evidence for consistent trends over time for the prevalence of children at, or below, the 2nd percentile. There is a lack of national data prior to, and beyond, the age of 4 years. Future work should focus on what is happening in early preschool years (0–3 years) as this time point has been reported to be one of the critical life periods for the development of obesity and a target for early intervention/prevention [55].

## Supplementary information

Supplementary information
