# Supplementary material for: Further reductions in the prevalence of obesity in 4-year-old New Zealand children from 2017 to 2019
Source: Int J Obes (Lond). 2022 Feb 25;46(6):1176–87. doi: 10.1038/s41366-022-01095-2 (PMC9151386; doi:10.1038/s41366-022-01095-2)
Supplement: Supplementary file 1 — Supplementary information [file 41366_2022_1095_MOESM1_ESM.docx]

**Supplementary Information**

**Supplement A** – Investigation of the reduction in mean BMI z-score for the latest two years of data (‘2017/18’ and ‘2018/19’)

**Supplement B** – Results for the prevalence of children at, or below, the 2^nd^ percentile

**Supplement A** – Investigation of the reduction in mean BMI z-score for the latest two years of data (‘2017/18’ and ‘2018/19’)

As shown in **Figure S.1.** there is a larger reduction in mean BMI z-score between ‘2016/17’ and ‘2017/18’ than we might expect, given the historical linear trend in BMI z-score.

**Figure S.1.** Changes in mean BMI z-score by fiscal year

Observed mean BMI z-score

Estimated mean BMI z-score based on linear trend calculated from ‘2011/12’ to ‘2016/17’ and forecast to ‘2018/19’

**Check 1: Investigate height, weight, and age data**

We set out to investigate whether there was evidence for increased errors in the height and weight data for the ‘2017/18’ and ‘2018/19’ fiscal years, or whether there were changes in the distribution of age (in months) of the children. **Table S.1.** shows descriptive statistics for height and weight. While there is a slight increase in mean height from ‘2016/17’ to ‘2017/18’ – mean height in ‘2017/18’ and ‘2018/19’ is similar to previous years (‘2013/14’ and ‘2014/15’) and the median is consistently 106cm across all years. The standard deviation, indicating the spread of the distribution, is similar across years ranging from 4.7-4.9. The extremes of the distribution (1^st^ and 99^th^ percentiles) are also very similar across all years. Therefore, there is little evidence that the height measures in ‘2017/18’ and ‘2018/19’ contain more error (random or systematic) than any other years.

**Table S.1.** Descriptive statistics for height, weight and age, by fiscal year

|  | **2011/12** | **2012/13** | **2013/14** | **2014/15** | **2015/16** | **2016/17** | **2017/18** | **2018/19** |
| --- | --- | --- | --- | --- | --- | --- | --- | --- |
|  | Height (cm) | | | | | | | |
| Mean | 106.3 | 106.4 | 106.3 | 106.0 | 105.9 | 105.9 | 106.0 | 106.1 |
| SD | 4.9 | 4.9 | 4.8 | 4.8 | 4.7 | 4.8 | 4.8 | 4.9 |
| Median | 106 | 106 | 106 | 106 | 106 | 106 | 106 | 106 |
| 1^st^ Percentile | 95.4 | 95.5 | 95.5 | 95.0 | 95.0 | 95.0 | 95.0 | 95.0 |
| 99^th^ Percentile | 118.0 | 118.4 | 118.0 | 117.8 | 117.5 | 118.0 | 118.0 | 118.0 |
|  | Weight (kg) | | | | | | | |
| Mean | 18.5 | 18.5 | 18.5 | 18.4 | 18.3 | 18.3 | 18.3 | 18.23 |
| SD | 2.8 | 2.7 | 2.7 | 2.7 | 2.7 | 2.7 | 2.7 | 2.7 |
| Median | 18.5 | 18.5 | 18.5 | 18.4 | 18.3 | 18.3 | 18.3 | 18.3 |
| 1^st^ Percentile | 13.5 | 13.4 | 13.4 | 13.3 | 13.4 | 13.4 | 13.2 | 13.2 |
| 99^th^ Percentile | 27.1 | 27.0 | 27.0 | 27.0 | 26.8 | 27.0 | 26.6 | 26.8 |
|  | Age (months)^a^ | | | | | | | |
| Mean | 52.4 | 52.4 | 52.3 | 51.9 | 51.7 | 51.7 | 51.6 | 51.7 |
| SD | 3.5 | 3.6 | 3.5 | 3.2 | 3.2 | 3.1 | 3.2 | 3.3 |
| Median | 51 | 51 | 51 | 51 | 51 | 51 | 50 | 51 |

^a^Age of the sample was restricted to those aged 48-60 months

Mean and median values for weight consistently decrease over time and the reductions in weight from ‘2016/17’ to ‘2017/18’ are in line with expectations based on the decreasing trend in weight over time (**Figure S.2.**). Standard deviation values for weight are similar across years ranging from 2.7-2.8. The extremes of the distribution (1^st^ and 99^th^ percentiles) have both decreased slightly over time – in keeping with a shifting distribution in weight. There is little evidence that the weight measures in ‘2017/18’ and ‘2018/19’ contain more error (random or systematic) than any other years.

**Figure S.2.** Mean weight by fiscal year

Observed mean weight

Estimated mean weight based on linear trend calculated from ‘2011/12’ to ‘2016/17’ and forecast to ‘2018/19’

As BMI changes with age, it is plausible that differences in age could lead to changes in BMI z-scores – even though these scores are standardized by age. We checked the average age of respondents in months for each year. While mean age was higher between ‘2011/12’ to ‘2013/14’ there is very little change in mean age from ‘2014/15’ onwards. Standard deviation also tended to be higher between ‘2011/12’ to ‘2013/14’, and the median was lower than the mean, suggesting a higher number of children measured at older ages in ‘2011/12’ and ‘2013/14’ compared to ‘2014/15’ onwards. Median age is consistently 51 months, apart from ‘2017/18’ where it is 50 months. There is little evidence for changes in ages contributing to the reduction in BMI z-score between ‘2016/17’ and ‘2018/19’.

**Check 2. Investigate consistency in the reduction in BMI z-scores across subgroups in the sample**

The larger reduction in BMI z-score between ‘2016/17’ and ‘2017/18’ was present for both male and female children, children of different ethnicities, children who lived in areas of high and low deprivation, and children who live in rural and urban areas (**Table S.2**.).

For male children mean BMI z-score decreased by 0.06 units and for female children BMI z-score decreased by 0.07 units. Decreases in mean BMI z-scores ranged from 0.05 to 0.11 units across major ethnic groups with the largest decrease for Pasifika children. **Figure S.3.** presents changes over time in mean BMI z-score for Māori, Pacific and Asian children. Within Pasifika subgroups decreases were largest for Tongan (BMI z-score decreased by 0.16 units) and Cook Island Māori children (BMI z-score decreased by 0.14 units) and were smaller for Samoan (BMI z-score decreased by 0.08 units) and other pacific groups (BMI z-score decreased by 0.09 units). Mean BMI z-score decreased by 0.07 units for children residing in urban areas, and 0.05 for children residing in rural areas. Across deprivation quintiles decreases in BMI z-scores ranged from 0.06 to 0.10 units.

**Table S.2.** Change in BMI z-score between ‘2016/17’ and ‘2017/18’, by subgroup

|  | **2016/17** | **2017/18** | **Δ ^a^** |
| --- | --- | --- | --- |
| Overall | 0.64 | 0.57 | 0.07 |
| Sex |  |  |  |
| Male | 0.70 | 0.64 | 0.06 |
| Female | 0.57 | 0.50 | 0.07 |
| Ethnicity |  |  |  |
| European | 0.63 | 0.58 | 0.05 |
| Māori | 0.86 | 0.78 | 0.08 |
| Pacific | 1.10 | 0.99 | 0.11 |
| Samoan | 1.16 | 1.08 | 0.08 |
| Tongan | 1.26 | 1.10 | 0.16 |
| Cook Island Māori | 1.01 | 0.87 | 0.14 |
| Other Pacific | 1.01 | 0.92 | 0.09 |
| Asian | 0.25 | 0.16 | 0.09 |
| Area |  |  |  |
| Urban^b^ | 0.63 | 0.56 | 0.07 |
| Rural^c^ | 0.67 | 0.62 | 0.05 |
| Deprivation^d^ |  |  |  |
| NZDep Q1 | 0.50 | 0.44 | 0.06 |
| NZDep Q2 | 0.54 | 0.46 | 0.08 |
| NZDep Q3 | 0.60 | 0.53 | 0.07 |
| NZDep Q4 | 0.66 | 0.60 | 0.06 |
| NZDep Q5 | 0.84 | 0.74 | 0.10 |

^a^Change over time, between ‘2016/17’ and ‘2017/18’

^b^Includes major, secondary and minor urban areas with populations >1 000.

^c^Includes areas with populations <999.

^d^Household deprivation categorised using NZDep scale, quintile 1 indicates the lowest level of deprivation and quintile 5 indicates the highest level of deprivation (1).

**Figure S.3.** Changes in mean BMI z-score for Māori, Pacific and Asian children, by fiscal year


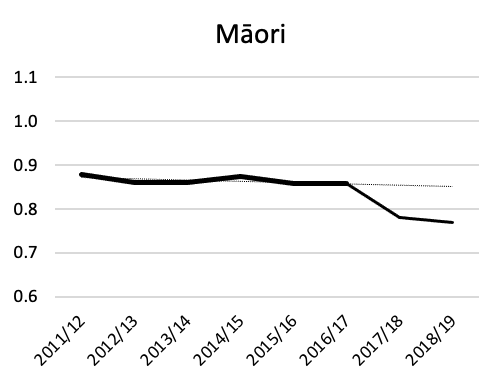

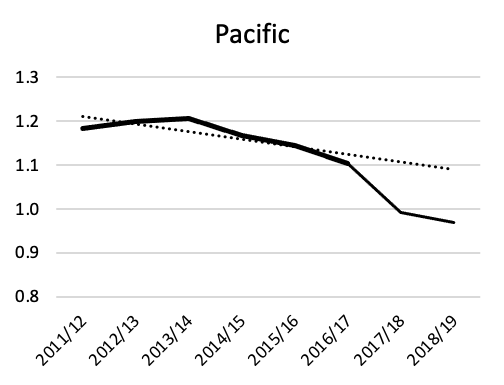

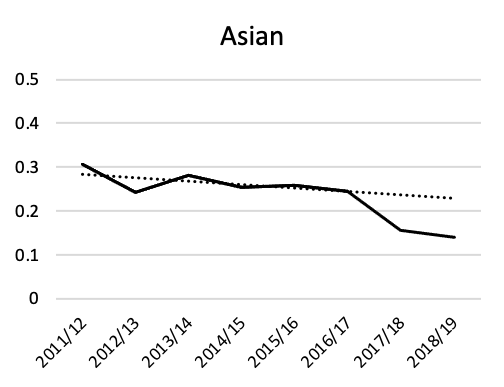


**BMI z-score**

As with the previous graphs presented, the dashed line represents the estimated mean BMI z-score based on linear trend calculated from ‘2011/12’ to ‘2016/17’ and forecast to ‘2018/19’ and the black solid line represents the observed mean BMI z-score for each group.

Note: the Y axis is different on these three graphs due to the large differences in BMI z-score by ethnicity.

**BMI z-score**

**Supplement B**. Results for the prevalence of children at, or below, the 2^nd^ percentile

**Figure S.4.** Trends in the percentage of children at, or below, the 2^nd^ percentile, overall and by sociodemographic characteristics


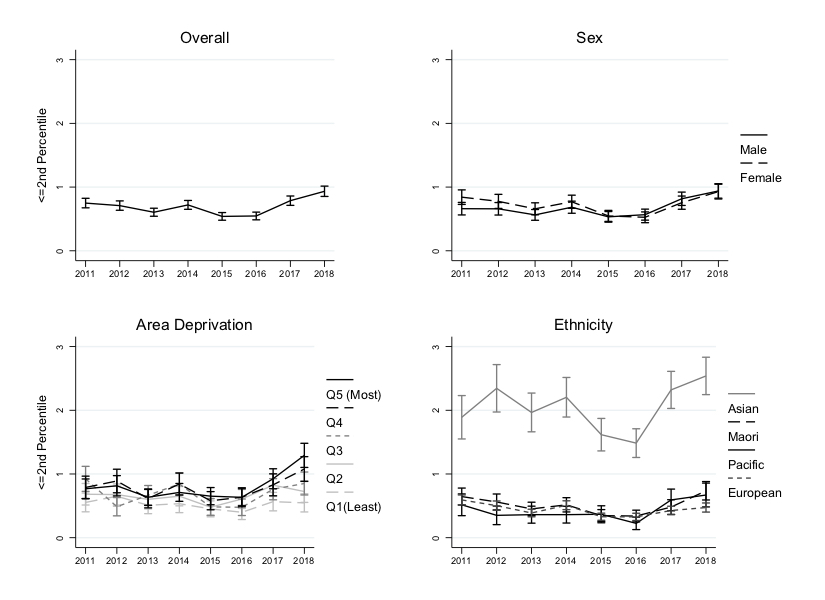


**Table S.3**. Year specific percentage of children at, or below the 2^nd^ percentile of age- and sex-adjusted BMI by sociodemographic characteristics

| <2^nd^ Percentile | 2011/12 | 2012/13 | 2013/14 | 2014/15 | 2015/16 | 2016/17 | 2017/18 | 2018/19 |
| --- | --- | --- | --- | --- | --- | --- | --- | --- |
| Overall | 0.75  (0.67, 0.82) | 0.71  (0.64, 0.78) | 0.61  (0.54, 0.67) | 0.72  (0.65, 0.79) | 0.54  (0.48, 0.60) | 0.55  (0.49, 0.61) | 0.79  (0.71, 0.86) | 0.93  (0.85, 1.02) |
| Male | 0.66  (0.56, 0.76) | 0.66  (0.56, 0.76) | 0.56  (0.48, 0.65) | 0.68  (0.59, 0.78) | 0.53  (0.45, 0.62) | 0.57  (0.48, 0.65) | 0.82  (0.71, 0.92) | 0.94  (0.83, 1.05) |
| Female | 0.84  (0.73, 0.96) | 0.78  (0.67, 0.89) | 0.66  (0.57, 0.75) | 0.77  (0.67, 0.87) | 0.55  (0.46, 0.63) | 0.53  (0.44, 0.61) | 0.76  (0.65, 0.86) | 0.93  (0.81, 1.05) |
| European | 0.59  (0.52, 0.67) | 0.50  (0.43, 0.58) | 0.39  (0.33, 0.45) | 0.51  (0.44, 0.58) | 0.33  (0.27, 0.39) | 0.33  (0.27, 0.38) | 0.43  (0.36, 0.49) | 0.47  (0.40, 0.55) |
| Māori | 0.65  (0.51, 0.78) | 0.56  (0.44, 0.69) | 0.45  (0.35, 0.56) | 0.52  (0.40, 0.63) | 0.35  (0.25, 0.44) | 0.34  (0.25, 0.43) | 0.48  (0.37, 0.60) | 0.74  (0.59, 0.88) |
| Pacific | 0.52  (0.35, 0.69) | 0.35  (0.21, 0.50) | 0.36  (0.23, 0.50) | 0.36  (0.23, 0.49) | 0.37  (0.23, 0.50) | 0.23  (0.13, 0.32) | 0.59  (0.42, 0.76) | 0.67  (0.48, 0.86) |
| Asian | 1.89  (1.55, 2.23) | 2.35  (1.97, 2.72) | 1.97  (1.66, 2.27) | 2.20  (1.89, 2.52) | 1.62  (1.36, 1.87) | 1.49  (1.26, 1.71) | 2.32  (2.03, 2.61) | 2.54  (2.25, 2.83) |
| Urban^a^ | 0.78  (0.70, 0.86) | 0.74  (0.66, 0.82) | 0.63  (0.56, 0.70) | 0.79  (0.71, 0.87) | 0.55  (0.49, 0.62) | 0.57  (0.50, 0.64) | 0.84  (0.76, 0.92) | 1.01  (0.92, 1.10) |
| Rural^b^ | 0.48  (0.30, 0.65) | 0.46  (0.30, 0.62) | 0.42  (0.26, 0.57) | 0.29  (0.17, 0.42) | 0.42  (0.27, 0.57) | 0.39  (0.24, 0.54) | 0.46  (0.30, 0.61) | 0.43  (0.27, 0.59) |
| Deprivation^c^ Q1 | 0.55  (0.41, 0.70) | 0.65  (0.49, 0.81) | 0.51  (0.38, 0.64) | 0.53  (0.39, 0.68) | 0.45  (0.33, 0.58) | 0.40  (0.29, 0.51) | 0.57  (0.42, 0.71) | 0.55  (0.40, 0.69) |
| Deprivation^c^ Q2 | 0.68  (0.51, 0.85) | 0.68  (0.51, 0.84) | 0.60  (0.45, 0.75) | 0.65  (0.50, 0.80) | 0.48  (0.35, 0.61) | 0.61  (0.46, 0.76) | 0.83  (0.65, 1.00) | 0.73  (0.55, 0.90) |
| Deprivation^c^ Q3 | 0.92  (0.73, 1.12) | 0.49  (0.34, 0.63) | 0.67  (0.51, 0.82) | 0.84  (0.67, 1.02) | 0.48  (0.35, 0.62) | 0.48  (0.35, 0.61) | 0.76  (0.60, 0.93) | 0.85  (0.67, 1.03) |
| Deprivation^c^ Q4 | 0.79  (0.61, 0.97) | 0.89  (0.71, 1.08) | 0.61  (0.47, 0.76) | 0.84  (0.67, 1.01) | 0.58  (0.44, 0.72) | 0.64  (0.49, 0.78) | 0.83  (0.66, 1.00) | 1.08  (0.88, 1.27) |
| Deprivation^c^ Q5 | 0.77  (0.62, 0.92) | 0.82  (0.66, 0.97) | 0.64  (0.51, 0.76) | 0.71  (0.57, 0.85) | 0.65  (0.52, 0.79) | 0.63  (0.50, 0.77) | 0.93  (0.77, 1.08) | 1.29  (1.10, 1.48) |

Percentages expressed as mean (95% CI).

^a^Includes major, secondary and minor urban areas with populations >1 000.

^b^Includes areas with populations <999.

^c^Household deprivation categorised using NZDep scale, quintile 1 indicates the lowest level of deprivation and quintile 5 indicates the highest level of deprivation (1).

**References**

1. Atkinson J, Salmond C, Crampton P. NZDep2018 Index of Deprivation User's Manual [Internet]. Wellington; 2019 [cited 2021 Jul 16]. Available from: https://www.otago.ac.nz/wellington/otago730391.pdf
